# Alteration of calcium signalling in cardiomyocyte induced by simulated microgravity and hypergravity

**DOI:** 10.1111/cpr.12783

**Published:** 2020-02-26

**Authors:** Caizhi Liu, Guohui Zhong, Yuezhang Zhou, Yuchen Yang, Yingjun Tan, Yuheng Li, Xingcheng Gao, Weijia Sun, Jianwei Li, Xiaoyan Jin, Dengchao Cao, Xinxin Yuan, Zizhong Liu, Shuai Liang, Youyou Li, Ruikai Du, Yinlong Zhao, Jianqi Xue, Dingsheng Zhao, Jinping Song, Shukuan Ling, Yingxian Li

**Affiliations:** ^1^ State Key Lab of Space Medicine Fundamentals and Application China Astronaut Research and Training Center Beijing China; ^2^ Beijing National Day School Beijing China; ^3^ State Key Laboratory of Agrobiotechnology College of Life Sciences China Agricultural University Beijing China; ^4^ Xiyuan Hospital China Academy of Chinese Medical Sciences Beijing China; ^5^ Key Laboratory of Molecular and Cellular Biology of Ministry of Education College of Life Science Hebei Normal University Shijiazhuang China

**Keywords:** Ca^2+^, cardiac remodelling, hypergravity, microgravity, proliferation

## Abstract

**Objectives:**

Cardiac Ca^2+^ signalling plays an essential role in regulating excitation‐contraction coupling and cardiac remodelling. However, the response of cardiomyocytes to simulated microgravity and hypergravity and the effects on Ca^2+^ signalling remain unknown. Here, we elucidate the mechanisms underlying the proliferation and remodelling of HL‐1 cardiomyocytes subjected to rotation‐simulated microgravity and 4G hypergravity.

**Materials and Methods:**

The cardiomyocyte cell line HL‐1 was used in this study. A clinostat and centrifuge were used to study the effects of microgravity and hypergravity, respectively, on cells. Calcium signalling was detected with laser scanning confocal microscopy. Protein and mRNA levels were detected by Western blotting and real‐time PCR, respectively. Wheat germ agglutinin (WGA) staining was used to analyse cell size.

**Results:**

Our data showed that spontaneous calcium oscillations and cytosolic calcium concentration are both increased in HL‐1 cells after simulated microgravity and 4G hypergravity. Increased cytosolic calcium leads to activation of calmodulin‐dependent protein kinase II/histone deacetylase 4 (CaMKII/HDAC4) signalling and upregulation of the foetal genes *ANP* and *BNP*, indicating cardiac remodelling. WGA staining indicated that cell size was decreased following rotation‐simulated microgravity and increased following 4G hypergravity. Moreover, HL‐1 cell proliferation was increased significantly under hypergravity but not rotation‐simulated microgravity.

**Conclusions:**

Our study demonstrates for the first time that Ca^2+^/CaMKII/HDAC4 signalling plays a pivotal role in myocardial remodelling under rotation‐simulated microgravity and hypergravity.

## 1 INTRODUCTION

Altered gravity conditions, such as micro‐ and hypergravity, have different effects on living beings at various levels of organization, including changing the biophysical properties of a single cell up to the level of the entire organism.^1^, ^2^, ^3^, ^4^, ^5^ The human cardiovascular system has adapted to the 1G gravity on Earth. Changes in gravity can modulate the structure and morphology of the heart. Exposure to the microgravity environment of space leads to cardiac atrophy and a decline in cardiac function. Studies of head‐down‐tilt bed rest, shown to be a useful and reliable model for many of the physiological effects induced by human spaceflight, have demonstrated that the human heart atrophies at a rate of approximately 1% per week in the absence of countermeasures.^6^, ^7^ Goldstein et al^8^ found that the cross‐sectional area of myofibrils of papillary and ventricular muscles was decreased, and the myocardium had atrophied in rats after space flight. Hindlimb unloading (HU) of rodents has been used as a ground‐based model to mimic the effects of microgravity. Our previous study demonstrated that the phosphorylation levels of histone deacetylase 4 (HDAC4) were increased in the hearts of mice after 28 days of HU‐simulated microgravity. Phosphorylation of HDAC4 causes its relocalization to the cytoplasm and activation of myocyte enhancer factor 2 (MEF2) and cardiac remodelling genes, such as atrial natriuretic peptide (*ANP*) and brain natriuretic peptide (*BNP*) in cardiomyocytes.^9^, ^10^, ^11^ Ca^2+^/calmodulin‐dependent protein kinase II (CaMKII) activates transcriptional regulators directly by phosphorylating HDAC4.

Intracellular Ca^2+^ levels also play an important role in the regulation of cardiac remodelling. Ca^2+^ functions through the Ca^2+^ binding protein calmodulin (CaM) to activate CaMKII, which is activated by different pathological processes in the heart. This Ca^2+^‐CaMKII‐dependent gene regulation during cardiac remodelling suggests novel strategies for Ca^2+^‐CaMKII‐dependent “transcriptional therapies” to control cardiac gene expression and function.^9^ In general, much less is known about the effects of hypergravity on the heart. In one study, heart mass was significantly increased in hypergravity‐exposed mice compared with a 1 G control group,^12^ and our previous study indicated that hypergravity induced differentiation of bone marrow mesenchymal stem cells into cardiomyocytes.^13^ However, with the exception of these studies, little is known of the mechanisms regulating the alteration of cardiomyocytes induced by hypergravity.

Ca^2+^ is a highly versatile intracellular signal that regulates many different cellular processes.^14^ Dynamic cardiac Ca^2+^ signalling plays an essential role in regulating cardiac functions, including cardiac contraction, relaxation and remodelling.^15^ Under conditions of microgravity, urinary calcium excretion is increased, intestinal calcium absorption is decreased, serum calcium is increased, and the overall calcium balance in the body is disrupted.^16^, ^17^ Microgravity can also lead to disrupted calcium homoeostasis in cardiovascular cells.^18^ Mice subjected to HU by tail suspension for 28 days exhibited abnormal intracellular Ca^2+^ handling in cardiomyocytes. HU of rats impaired the function of L‐type Ca^2+^ channels and decreased intracellular Ca^2+^ ([Ca^2+^]_i_) transients, resulting in reduced responsiveness to β‐adrenoceptor stimulation, which may be partially responsible for the decline in cardiac function. These studies demonstrated that microgravity‐induced changes in Ca^2+^ signalling play an important role in cardiac remodelling and reduced function. Numerous studies on mammalian organisms have demonstrated that the absence of gravity has severe effects not only on a systemic level but also on a cellular level.^2^, ^19^, ^20^, ^21^, ^22^, ^23^ However, the changes in intracellular calcium signalling, and its regulatory role in cardiac remodelling under altered gravity, are not fully understood.

In vitro studies have demonstrated that space flight and simulated microgravity induce significant changes in gene expression patterns,^24^, ^25^, ^26^ autophagy,^27^ cell migration,^28^, ^29^ extracellular matrix composition^30^ and the cytoskeleton.^31^ The clinostat is widely used for space biology research, as it can simulate the effect of microgravity on cells.^32^, ^33^, ^34^, ^35^ Although HL‐1 cells derive from atrial myocytes, they maintain the ability to contract and retain differentiated cardiac morphological, biochemical and electrophysiological properties.^36^ HL‐1 cells have thus proven useful as a model for studying contracting cardiomyocytes, because of their organized structure and ability to contract in culture^37^; HL‐1 cells have been used in many studies of myocardial remodelling.^38^, ^39^, ^40^, ^41^ Here, we report that calcium signalling plays a pivotal role in regulating gravity alteration‐induced cardiac remodelling through the Ca^2+^/CaMKII/HDAC4 signalling pathway.

## 2 MATERIALS AND METHODS

### 2.1 Cell culture

The HL‐1 cardiomyocyte cell line was cultured with Claycomb medium (Sigma‐Aldrich), 10% foetal bovine serum (Gibco), 1% norepinephrine, 1% penicillin‐streptomycin and 1% l‐glutamine (Sigma‐Aldrich) in a 5% CO_2_ atmosphere. Cells were seeded in T‐75 culture plates precoated with 25 µg/mL fibronectin solution.

### 2.2 Rotation‐simulated microgravity

**Figure 1:**
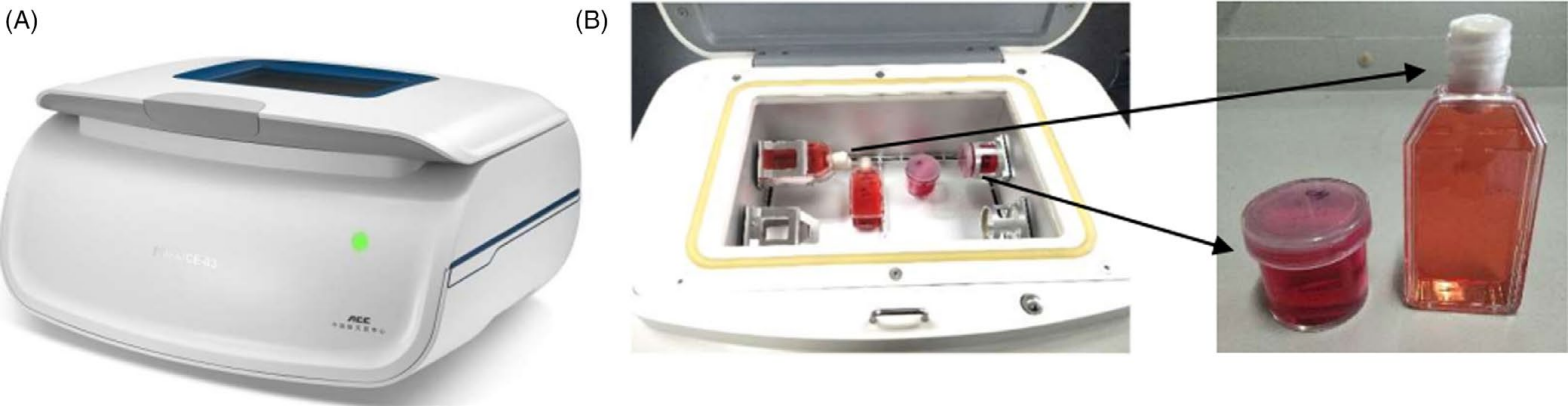
The clinostat developed and provided by the China Astronaut Research and Training Center. A, Clinostat device. B, Internal composition of the clinostat with cell culture flasks. The columnar bottle or cell culture flasks were filled with culture medium

To simulate the effects of microgravity, we used a two‐dimensional (2D) clinostat, which was developed and provided by the China Astronaut Research and Training Center (Figure 1A). HL‐1 cells were incubated in 25 cm^2^ cell culture flasks or plated on 25‐mm glass coverslips and filled with culture medium (Figure 1B). To avoid the influence of shear stress, all culture flasks were filled with medium to eliminate air bubbles and hermetically closed during rotation. The cells were rotated around a horizontal axis at a speed of 30 rpm, which resulted in randomization of the gravitational vector. It was equivalent to the microgravity of low earth orbit (about 0.01 g). The control group was cultured in the same manner as the experimental group, but without clinorotation. The 2D clinostat has been described previously.^42^

### 2.3 Hypergravity centrifuge

A hypergravity centrifuge was used to detect the effects of hypergravity on cells. The details of the procedure were the same as described above for rotation‐simulated microgravity. The hypergravity centrifuge was continuously rotated under the 4G hypergravity condition at 37°C for 48 hours. The hypergravity centrifuge has been described previously.^13^

### 2.4 Measurement of intracellular Ca^2+^

A calcium indicator was used to measure intracellular Ca^2+^ levels and dynamic calcium signalling, as described previously.^43^ Briefly, the coverslip was transferred to the chamber and cells were loaded with 5 μmol/L Fluo‐4 AM (Molecular Probes) for 20 minutes at 37°C in Tyrode solution (137 mmol/L NaCl, 20 mmol/L HEPES, 10 mmol/L glucose, 1.2 mmol/L MgCl_2_·6H_2_O, 1.2 mmol/L NaH_2_‐PO_4_·2H_2_O, 5.4 mmol/L KCl and 1.8 mmol/L CaCl_2_, pH 7.35 [adjusted with NaOH]). Cells were then rinsed twice with Tyrode solution and mounted on the inverted stage of a confocal microscope. Confocal imaging was performed with an LSM710 microscope (Zeiss) with a 40×, 1.3 NA oil immersion objective, and linescan speed of 2.55 μs/line; the pinhole was nominally set for a 1‐μm optical section. Fluo‐4 AM was excited at 488 nm, and fluorescence emission was measured at 490‐550 nm. Images were acquired every 3 seconds to observe calcium oscillations and analysed using Interactive Data Language (IDL: Research Systems) software. The fluorescence intensity was quantified, and the calcium oscillation trace was acquired. We took the number of oscillations in 1 minute to be the calcium oscillation frequency. To determine resting [Ca^2+^]_i_ and [Ca^2+^]_i_ released from the endoplasmic reticulum (ER), cells were scanned for 20‐30 seconds to obtain F_resting_; then, the solution was replaced with 0 Ca^2+^ Tyrode solution containing 4 mmol/L EGTA (Invitrogen), 5 μmol/L thapsigargin (Molecular Probes) and 10 μmol/L A23187 (Sigma‐Aldrich). Stored calcium was immediately released to the cytoplasm, followed by a gradual decline; we defined the peak value as F_ER release_. When stabilized, 100 μmol/L BAPTA‐AM (Molecular Probes) was added to further chelate cytosolic Ca^2+^, to measure the minimum fluorescence level (F_min_). Then, the solution was replaced with Tyrode solution containing 10 mmol/L Ca^2+^, 5 μmol/L thapsigargin, 12 μmol/L A23187, 3 μmol/L FCCP (Sigma‐Aldrich) and 20 mmol/L 2‐DG (Sigma‐Aldrich). The stable value was F_max_. The trace is shown in Figure 1A. Finally, [Ca^2+^]_i_ was calibrated using a modified version of the equation of Grynkiewicz et al^44^:

$$\left[{\text{Ca}}^{2+}\right]=K_{d}\times \left(F-F_{\min}\right)/\left(F_{\max}-F\right).$$

### 2.5 Protein extraction and Western blot

HL‐1cells were lysed in lysis buffer (50 mmol/L Tris, pH 7.5, 250 mmol/L NaCl, 0.1% sodium dodecyl sulphate, 2 mmol/L dithiothreitol, 0.5% NP‐40, 1 mmol/L PMSF and protease inhibitor cocktail) on ice for 30 minutes. Protein fractions were collected by centrifugation at 15 000 *g* at 4°C for 30 minutes. Protein samples were separated by 10% SDS–PAGE and transferred to polyvinylidene difluoride (PVDF) membranes. The membranes were blocked with 5% bovine serum albumin and incubated with specific antibodies overnight. Antibodies used were as follows: CaMKII (1:1000, GeneTex, GTX111401), p‐CaMKII (1:1000, T287, GeneTex, GTX52342), HDAC4 (1:1000, Cell Signalling Technology, #5392), p‐HDAC4 (1:1000, S632, Cell Signalling Technology, #3424), mTOR (1:1000, Cell Signalling Technology, #2972), p‐mTOR (1:1000, Cell Signalling Technology, #2971), PCNA (1:1000, Cell Signalling Technology, #13110), α‐MHC (1:1000, Abclonal) and GAPDH (1:5000, Abways Technology, AB0036).

### 2.6 RNA extraction and real‐time PCR

Total RNA from HL‐1 cells was extracted with TRIzol Reagent (Invitrogen) as the manufacturer's instructions. RNA (0.5 μg) was reverse transcribed with PrimeScript RT reagent Kit (TaKaRa) according to the manufacturer's instructions. cDNA was used for detecting mRNA expression by quantitative PCR using SYBR^®^ Premix Ex TaqTMII Kit (TaKaRa). Primers used in this study were as follows:

**Table:** 

*ANP*(NM_008725)	Forward primer 5′‐TTCGGGGGTAGGATTGACAG‐3′, reverse primer 5′‐CACACCACAAGGGCTTAGGA‐3′, product length 142 bp
*BNP*(NM_001287348)	Forward primer 5′‐TGTTTCTGCTTTTCCTTTATCTG‐3′, reverse primer 5′‐TCTTTTTGGGTGTTCTTTTGTGA‐3′, product length 182 bp
*α‐MHC*(NM_001164171)	Forward primer 5′‐CCTCAAGCTCATGGCTACAC‐3′, reverse primer 5′‐TTGCCTCCTTTGCCTTTACC‐3′, product length 78 bp
*Gapdh*(NM_001289726)	Forward primer 5′‐ACTCCACTCACGGCAAATTCA‐3′, reverse primer 5′‐GGCCTCACCCCATTTGATG‐3′, product length 122 bp
*PCNA*(NM_011045)	Forward primer 5′‐AAGGGCTGAAGATAATGCAGAC‐3′, reverse primer 5′‐GTGGCTAAGGTCTCGGCATA‐3′, product length 190 bp
*C‐fos*(NM_010234)	Forward primer 5′‐GGGACAGCCTTTCCTACTACC‐3′, reverse primer 5′‐AGATCTGCGCAAAA GTCCTG‐3′, product length 88 bp
*CyclinD1*(NM_007631)	Forward primer 5′‐CTGACAACTCTATCCGGCCC‐3′, reverse primer 5′‐TTGTTCTCATCCGCCTCTGG‐3′, product length 142 bp

### 2.7 Cell transfection

Cells were plated on 25‐mm glass coverslips and transfected with CaMKII small interfering RNA (siRNA) or negative control (NC) at 70% confluence, using Lipofectamine RNAiMAX in OptiMEM according to the manufacturer's instructions (Invitrogen). The NC siRNA sequence was 5′‐UUCUCCGAACGUGUCACGUTT‐3′, and the CaMKII siRNA sequence was 5′‐UCUAGAAUCUGUUGUAUACAA‐3′.

### 2.8 Immunofluorescence staining

HL‐1 cells were fixed with 4% paraformaldehyde for 15 minutes, washed twice with phosphate‐buffered saline (PBS) and incubated with wheat germ agglutinin (WGA; Vector Laboratories) at room temperature for 30 minutes. The cells were then washed with PBS and incubated with Hoechst (Invitrogen) for 3 minutes. Images were acquired using a confocal microscope (LSM 710; Zeiss). Cell size analysis was performed with ImageJ software (NIH).

### 2.9 Statistical analysis

All quantitative data are presented as the mean ± standard error of the mean. Data were generated from three independent replicates. Statistical differences among groups were analysed by one‐way analysis of variance (ANOVA) with a post hoc test applied. All statistical analyses were performed with Prism software (version 6.0; GraphPad Software Inc). Statistical significance was evaluated using unpaired Student's *t* test or one‐way ANOVA for multiple samples. Differences were considered significant at *P < .05, **P < .01, and ***P < .001.

## 3 RESULTS

### 3.1 Rotation‐simulated microgravity inducted altered spontaneous calcium signalling

**Figure 2:**
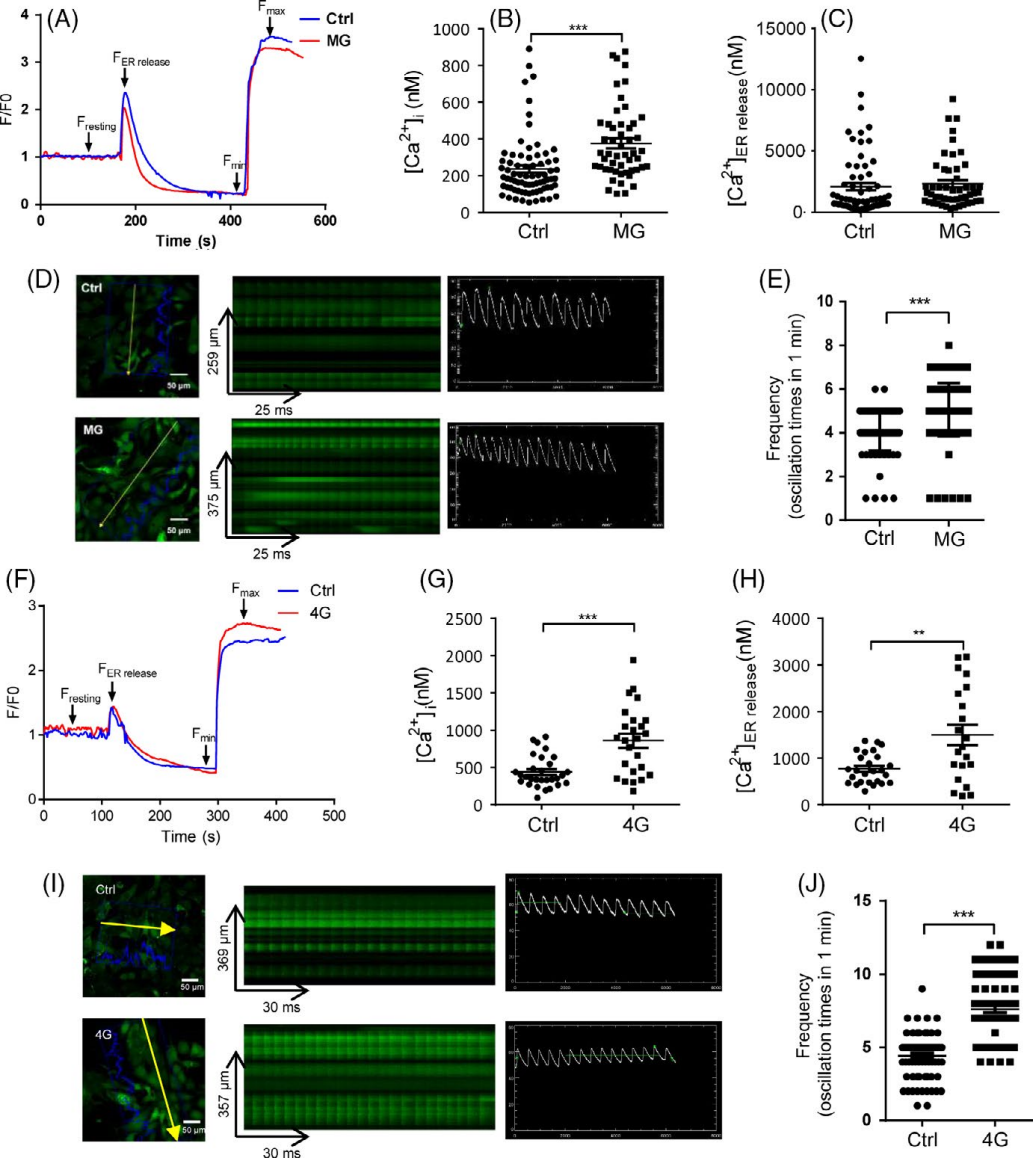
Altered spontaneous calcium signalling of HL‐1 cells following rotation‐simulated microgravity or hypergravity. A, Resting [Ca^2+^]_i_ and [Ca^2+^] released from the endoplasmic reticulum (ER) of HL‐1 cells in the rotation‐simulated microgravity (MG) and control (Ctrl) groups, shown by imaging cellular Ca^2+^ signals with Fluo‐4 AM. B, Basal cytosolic Ca^2+^ levels ([Ca^2+^]_i_) in HL‐1 cells in the presence or absence of MG (n = 74 [Ctrl] and n = 51 [MG]). Cells were pooled from three independent experiments. C, Ca^2+^ releases from the ER ([Ca^2+^]_ER release_) in HL‐1 cells in the control and MG groups (n = 66 [Ctrl] and n = 50 [MG]). Cells were pooled from three independent experiments. D, Ca^2+^ transients in HL‐1 cells in the presence or absence of MG, detected by line scanning with a confocal microscope. Scale bar: 50 μm. E, Chart shows the frequency of spontaneous calcium oscillations, which were detected by frame scanning with a confocal microscope (n = 106 [Ctrl] and n = 124 [MG]). F, Measurement of [Ca^2+^]_i_ and [Ca^2+^]_ER release_ in the 4G hypergravity and control groups. G, [Ca^2+^]_i_ in HL‐1 cells in the presence or absence of 4G hypergravity (n = 29 [Ctrl] and n = 25 [4G]). Cells were pooled from three independent experiments. H, [Ca^2+^]_ER release_ in HL‐1 cells from the control and 4G hypergravity groups (n = 26 [Ctrl] and n = 22 [MG]). Cells were pooled from three independent experiments. I, Ca^2+^ transients in HL‐1 cells in the presence or absence of 4G hypergravity, detected by line scanning with a confocal microscope. Scale bar: 50 μm. J, Chart shows the frequency of spontaneous calcium oscillations, detected by frame scanning with a confocal microscope (n = 77 [Ctrl] and n = 76 [4G]). Representative results of three independent experiments are shown. Data are shown as mean ± standard error of the mean (SEM); unpaired Student's *t* test, **P < .01 and ***P < .001

To determine the changes in intracellular calcium signalling under rotation‐simulated microgravity, HL‐1 cells were loaded with the cytosolic Ca^2+^‐sensitive fluorescent indicator Fluo‐4 AM, after 48 hours of rotation‐simulated microgravity with the 2D clinostat (Figure 1A). First, we detected the basal cytosolic Ca^2+^ ([Ca^2+^]_i_) and Ca^2+^ released from the ER ([Ca^2+^]_ER release_), which indicated the levels of calcium in the cytoplasm and ER, respectively (Figure 2A). [Ca^2+^]_i_ was increased significantly in HL‐1 cells under rotation‐simulated microgravity (Figure 2B). However, there was no difference in [Ca^2+^]_ER_ _release_ between the rotation‐simulated microgravity and control groups (Figure 2C). Cardiomyocytes are excitable cells that can produce spontaneous calcium oscillations. Using the line scan mode of the confocal microscope, we observed strong calcium transients in HL‐1 cells; moreover, the transients were increased in cells under rotation‐simulated microgravity (Figure 2D). The results obtained in frame scan mode showed more spontaneous calcium oscillations in cells under rotation‐simulated microgravity (Figure 2E) compared with the control group. These findings confirm that rotation‐simulated microgravity can promote intracellular calcium signalling.

### 3.2 Altered spontaneous calcium signalling was triggered by hypergravity

Astronauts experience hypergravity when travelling in space. We analysed intracellular calcium signalling in HL‐1 cells following centrifugation under the 4G hypergravity treatment. Basal cytosolic Ca^2+^ and Ca^2+^ released from the ER were measured (Figure 2F). [Ca^2+^]_i_ was increased significantly in HL‐1 cells under the 4G treatment (Figure 2G). In addition, more calcium was released from the ER in HL‐1 cells under 4G hypergravity, suggesting that ER Ca^2+^ stores are higher under conditions of hypergravity (Figure 2H). Using the line scan mode of the confocal microscope, we determined that calcium transients were increased in cells exposed to 4G hypergravity (Figure 2I); moreover, the frame scan mode showed more spontaneous calcium oscillations in these cells (Figure 2J). These observations indicate that intracellular and ER calcium levels are both increased markedly under 4G hypergravity.

### 3.3 Rotation‐simulated microgravity induced cardiomyocyte atrophy

**Figure 3:**
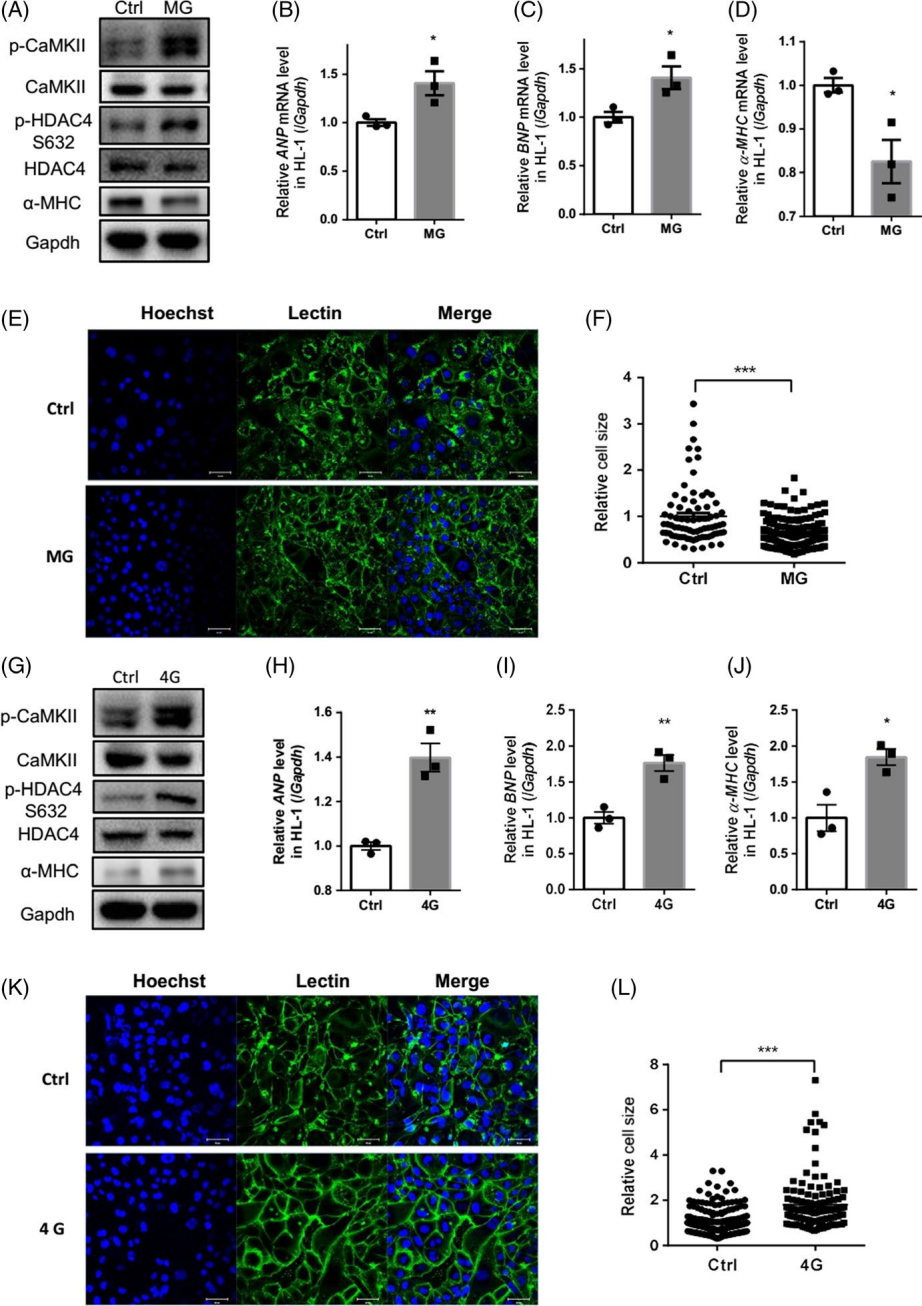
Rotation‐simulated microgravity and hypergravity activated cardiomyocyte remodelling. A, Expression of CaMKII and its phosphorylation at Thr287 (p‐CaMKII), HDAC4 and its phosphorylation at Ser632 (p‐HDAC4) and α‐MHC in HL‐1 cells. B‐D, mRNA levels of *ANP*, *BNP* and *α‐MHC* in HL‐1 cells. E and F, Wheat germ agglutinin (WGA) staining was used to demarcate the boundaries of HL‐1 cells following rotation for 48 h. The cell area was analysed and quantified. Scale bar: 50 μm (n = 78 [Ctrl] and n = 151 [MG]). G, Expression of p‐CaMKII, p‐HDAC4 and α‐MHC following 4G hypergravity. H‐J, Analysis of *ANP*, *BNP* and *α‐MHC* mRNA levels following 4G hypergravity. K and L, WGA staining was used to demarcate the boundaries of HL‐1 cells following 4G centrifugation for 48 h. The cell area was analysed and quantified. Scale bar: 50 μm (n = 256 [Ctrl] and n = 125 [4G]). CaMKII, calcium/calmodulin‐dependent protein kinase II; HDAC4, histone deacetylase 4; α‐MHC, myosin heavy chain α. *ANP*, atrial natriuretic peptide; *BNP*, brain natriuretic peptide. Representative results of three independent experiments are shown. Data are shown as mean ± SEM; unpaired Student's *t* test, *P < .05, **P < .01 and ***P < .001

The changes in cytosolic calcium concentration caused by calcium oscillations can encode complex and diverse signals, enabling calcium ions to regulate specific downstream pathways.^14^ To explore the effect of calcium oscillations on cardiomyocyte remodelling following clinostat‐simulated microgravity, changes in signalling associated with cardiomyocyte remodelling, and in embryonic gene expression, were assessed. Western blotting revealed that the phosphorylation of CaMKIIδ (Thr287) and HDAC4 (Ser632) in HL‐1 cells was increased significantly following rotation‐simulated microgravity (Figure 3A). Furthermore, treatment with siRNA‐CaMKII could inhibit the phosphorylation of HDAC4 (Figure S1A), indicating that the CaMKII/HDAC4 pathway is involved in simulated microgravity‐induced cardiac myocyte remodelling. Quantitative polymerase chain reaction (qPCR) revealed that expression of the foetal genes *ANP* and *BNP*, markers of myocardial remodelling, was significantly increased (Figure 3B,C). The level of myosin heavy chain α (α‐MHC) was decreased after 48 hours of rotation, indicating that clinostat‐simulated microgravity resulted in the activation of cardiomyocyte remodelling (Figure 3A,D).

Cell size was measured after 48 hours of rotation in the clinostat. WGA staining showed demarcation of cell boundaries, and cell size analysis revealed that HL‐1 cells were significantly smaller and atrophied after 48 hours of clinostat rotation (Figure 3E‐F); these effects could be prevented by treatment with siRNA‐CaMKII (Figure S1B). These results demonstrate that rotation‐simulated microgravity can lead to cardiomyocyte atrophy.

### 3.4 Hypergravity induced cardiomyocyte hypertrophy

Calcium signalling was increased in HL‐1 cells following exposure to hypergravity, which would affect downstream signalling. To explore the effect of calcium oscillations on cardiomyocyte remodelling signalling under conditions of hypergravity, we treated HL‐1 cells with 4G hypergravity for 48 hours, and cardiomyocyte remodelling signalling and foetal gene expression levels were analysed by Western blotting and qPCR, respectively. Phosphorylation of CaMKIIδ (Thr287) and HDAC4 (Ser632) was increased significantly after hypergravity (Figure 3G); these increases were inhibited by treatment with siRNA‐CaMKII (Figure S2A), demonstrating that the CaMKII/HDAC4 pathway was activated by hypergravity in cardiac myocytes. qPCR analysis showed that expression of the foetal genes *ANP* and *BNP* was increased significantly, indicating myocardial remodelling (Figure 3H,I). Expression of *α‐MHC* was also increased after 48 hours of hypergravity (Figure 3G,J), further suggesting that hypergravity resulted in the activation of signalling associated with cardiomyocyte remodelling.

To uncover the effects of hypergravity on cardiomyocytes, WGA staining was performed. HL‐1 cell size was increased significantly after 48 hours of hypergravity (Figure 3K,L), which could be prevented by siRNA‐CaMKII (Figure S2B). Thus, the CaMKII/HDAC4 pathway is also involved in hypergravity‐induced cardiac myocyte hypertrophy.

### 3.5 Rotation‐simulated microgravity did not affect the proliferation of HL‐1 cells

**Figure 4:**
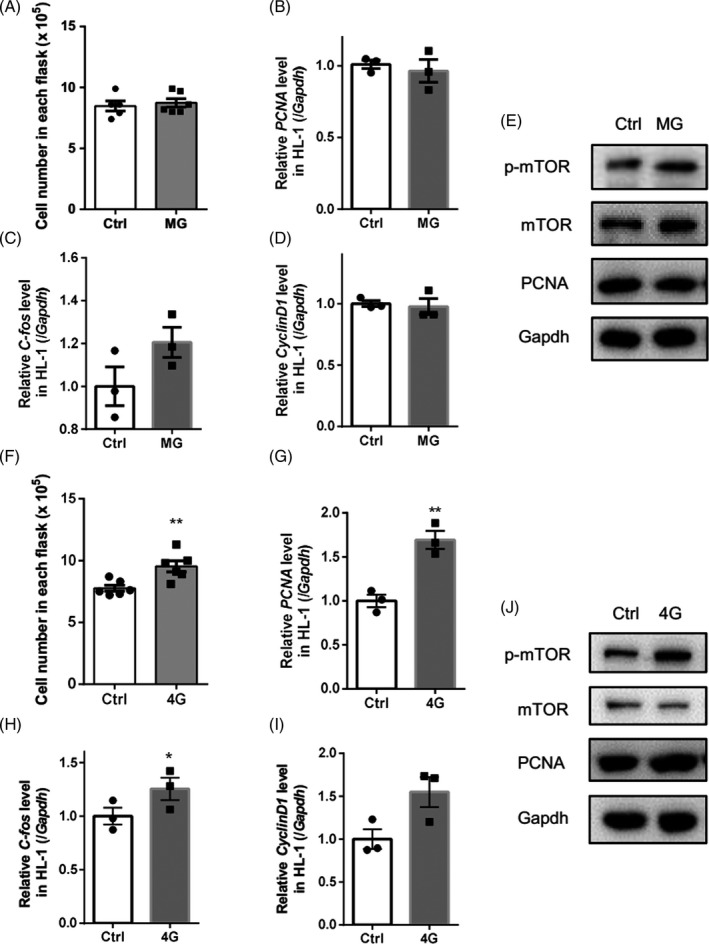
Effects of rotation‐simulated microgravity and hypergravity on HL‐1 cell proliferation. A, Analysis of cell number following microgravity. B‐D, mRNA levels of *PCNA*, *C‐fos* and *CyclinD1* were analysed by qPCR. E, Expression levels of mammalian target of rapamycin (mTOR), phosphorylated mTOR at Ser1248 (p‐mTOR) and PCNA in HL‐1 cells. F, Analysis of cell number following exposure to 4G hypergravity. G‐I, mRNA levels of *PCNA*, *C‐fos* and *CyclinD1* were analysed after 4G centrifugation for 48 h. J, Expression of p‐mTOR and PCNA in HL‐1 cells treated with 4G hypergravity. Representative results of three independent experiments are shown. Data are shown as mean ± SEM; unpaired Student's *t* test, *P < .05 and **P < .01

To determine the influence of microgravity on the proliferation of HL‐1 cells, cell count, Western blotting and qPCR analyses were performed to assess changes in proliferation‐related markers following rotation‐simulated microgravity. As shown in Figure 4A, compared with the control group, the cell number did not change in the microgravity group after 48 hours of rotation. The qPCR results showed that expression of the cell cycle marker genes *PCNA*, *CyclinD1* and *C‐fos* did not change following rotation (Figure 4B‐D). We also analysed changes in the phosphorylation of mammalian target of rapamycin (mTOR), which is involved in protein synthesis. The relative levels of phosphorylated mTOR (p‐mTOR)/mTOR and PCNA were also unchanged (Figure 4E), indicating that rotation‐simulated microgravity did not affect HL‐1 cell proliferation.

### 3.6 Hypergravity increased HL‐1 cell proliferation

As shown in Figure 4F, the cell number was increased significantly in the hypergravity group (Figure 4F). qPCR analysis showed that expression levels of the cell cycle marker genes *PCNA*, *CyclinD1* and *C‐fos* were also significantly increased following hypergravity treatment (Figure 4G‐I). We also analysed changes in mTOR phosphorylation and PCNA protein levels. The relative level of p‐mTOR/mTOR was increased significantly, indicating increased protein synthesis (Figure 4J). These results suggest that hypergravity increases the proliferation of HL‐1 cells.

## 4 DISCUSSION

This study showed that simulated microgravity and hypergravity could alter calcium signalling in cardiomyocytes. Spontaneous calcium oscillations and the cytosolic calcium concentration were both increased in HL‐1 cells after simulated microgravity and 4G hypergravity. Increased cytosolic calcium led to activation of the CaMKII/HDAC4 signalling pathway and upregulation of the cardiac foetal genes *ANP* and *BNP*. Cell size was decreased following rotation‐simulated microgravity and increased following 4G hypergravity. Moreover, HL‐1 cell proliferation was significantly increased with hypergravity, but not with rotation‐simulated microgravity. The changes in calcium signalling induced by altered gravity may be of functional significance during the cardiac stress response elicited by space flight and hypergravity.

Altered gravity conditions, including micro‐ and hypergravity, have different effects on living beings at various levels of organization, including changing biophysical properties at the level of a single cell up to the entire organism.^1^, ^4^, ^45^ According to our previous experiments on osteoblasts,^13^ osteoclasts^46^ and mesenchymal stem cells,^13^, ^47^ the effects of 48‐h exposure to microgravity and 4G hypergravity are significant. Xiong et al showed that rat cardiomyocytes cultured under simulated microgravity for 48 hours induced upregulation of inducible nitric oxide synthase.^48^ In this study, 48‐hour 4G hypergravity and rotation‐simulated microgravity were the altered gravity conditions.

The heart undergoes continual remodelling in response to fluctuations in functional demand. Pathological hemodynamic overloading (eg, hypertension and myocardial infarction)^49^, ^50^ and unloading (eg, prolonged bed rest and ventricular assist devices)^51^ induce pathological hypertrophy and atrophy, respectively. Although cardiac atrophy has distinct phenotypes compared with hypertrophy, it leads to a strikingly similar decline of cardiac function and upregulation of cardiac remodelling marker genes.^52^ Moreover, altered gravity affects the structure and morphology of heart tissue.^12^ As a consequence of hypergravity, heart mass was significantly increased in mice^12^ and rats.^53^ Changes in cardiac remodelling marker genes under hypergravity conditions have not been described to date. In the present study, rotation‐simulated microgravity and centrifuge‐induced hypergravity led to cardiomyocyte atrophy and hypertrophy, respectively. Both models induced increases in the foetal genes *ANP* and *BNP*, indicating a shift towards the foetal myocardial gene profile, which induces remodelling.

Well‐characterized signalling molecules that regulate cardiac remodelling include CaMKII and HDAC4.^11^, ^54^ CaMKII activation, and its ability to regulate class II histone deacetylases such as HDAC4 and their nuclear shuttling, represents a critical Ca^2+^‐dependent signalling circuit involved in cardiac hypertrophy and heart failure.^55^ We previously reported that both simulated microgravity and pressure overload (transverse aortic constriction) induced phosphorylation of HDAC4 and led to cardiac remodelling in mice.^11^, ^21^ Here, we found that the CaMKII/HDAC4 pathway was activated in HL‐1 cells under both simulated microgravity and hypergravity conditions, although more dramatically with hypergravity. Our study demonstrates that altered gravity can induce changes in CaMKII/HDAC4 signalling in cardiomyocytes in vitro.

Ca^2+^ serves as a central intracellular messenger in the heart. And intracellular Ca^2+^ handling also takes part in cardiac remodelling regulation.^56^ Ca^2+^ functions through Ca^2+^ binding protein calmodulin (CaM) to activate CaMKII.^57^ In cardiomyocytes, Ca^2+^ signals regulate contraction and also a host of other cellular processes including gene regulation, cellular growth and death.^58^, ^59^ Recent studies have uncovered that the magnitude and temporal signature of Ca^2+^ signals is critical, as is the cellular localization of these signals.^60^ Two types of Ca^2+^ channels, the voltage‐gated L‐type Ca^2+^ channels, which control Ca^2+^ influx elicited by action potentials, and ryanodine receptors (RyRs), which mediate Ca^2+^ release from intracellular stores, reside on the surface of cell membranes and sarcoplasmic reticulum (SR) membranes, respectively.^61^ Calcium oscillations are caused by the periodic uptake and release of Ca^2+^. The frequency of oscillations in [Ca^2+^]_i_ corresponds to the speed of cyclic release of Ca^2+^ from the SR and re‐uptake of Ca^2+^ by SERCA, a calcium uptake channel on the SR membranes.^62^ Increased levels of SERCA2b induce cell proliferation, while knockdown of SERCA2b reduces cell contractility.^63^ Compared with hypertrophy, there was no decrease in SERCA2 protein expression in atrophic heart, which might be one of the key differences between hypertrophy and atrophy.^64^ It is critical to understand the mechanisms regulating cardiac remodelling during microgravity induced myocardial atrophy and hypertrophy. Here, we showed that both rotation‐simulated microgravity and hypergravity induced the increase of [Ca^2+^]_i_ and calcium oscillation in HL‐1. The difference is that hypergravity induced a stronger increase of calcium signalling, and [Ca^2+^]_ER release_ increased in the group of hypergravity but not rotation‐simulated microgravity. Our understanding of this issue is incomplete, but it may be the cause of different follow‐up downstream phenotypes.

Besides, cell proliferation plays a major role in maintaining cardiomyocyte homoeostasis.^65^ Though adult cardiomyocyte has lost the ability to entry cell cycle, cardiomyocyte from embryonic and neonatal mammal is capable of proliferating.^66^ Prior experiments have shown that externally applied forces result in increased proliferation in an E‐cadherin force‐dependent manner.^67^ In this study, we demonstrated that simulated microgravity and hypergravity can influence the cardiomyocyte proliferation in different extent. Rotation‐simulated microgravity did not affect the proliferation of HL‐1; however, the cell proliferation marker genes, *PCAN* and *C‐fos* increased in hypergravity, and the level of p‐mTOR/mTOR increased too. This study indicated that hypergravity increased the proliferation and protein synthesis of HL‐1.

**Figure 5:**
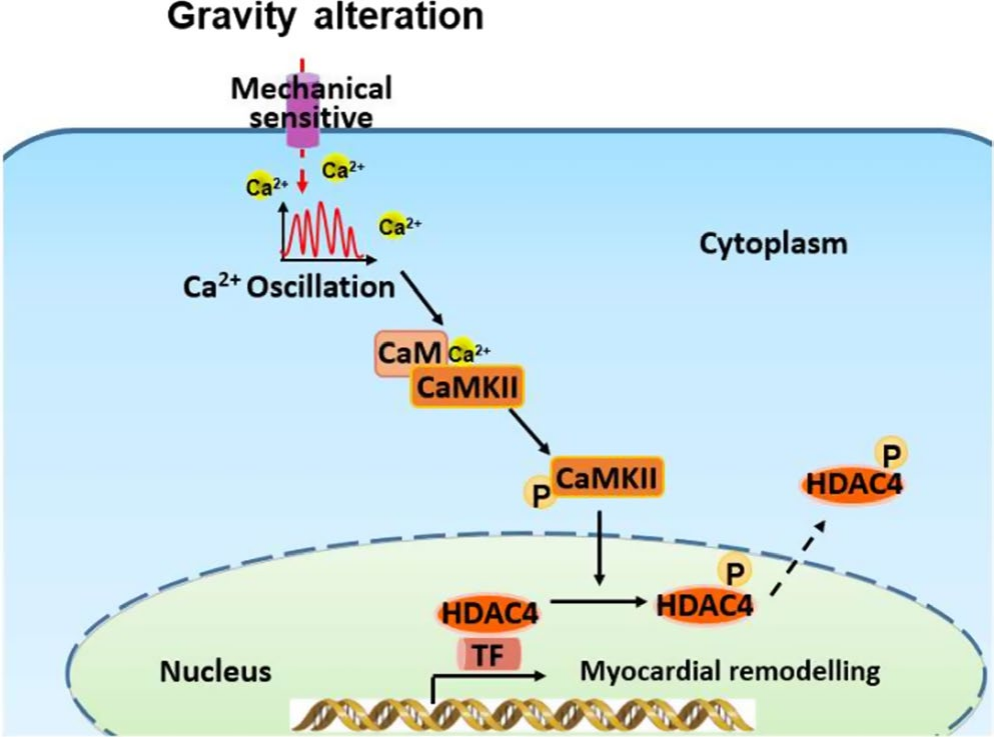
Schematic of the potential mechanism underlying the effects of gravity on myocardial remodelling. Alteration of microgravity induces dynamic changes in intracellular calcium signalling. The increase in [Ca^2+^]_i_ activates the CaMKII/HDAC4 signalling pathway and regulates myocardial remodelling. CaM, calmodulin; CaMKII, calmodulin‐dependent kinase type II; HDAC4, histone deacetylase 4; MEF2, myocyte enhancer factor 2; TF, transcription factor

We have reported for the first time that gravity induced changes in calcium signalling in HL‐1 cardiomyocytes (Figure 5); furthermore, these changes altered the response of HL‐1 cells to proliferation and remodelling. Consistent with previous reports, our study indicates that simulated microgravity leads to cardiomyocyte atrophy and that hypergravity promotes the proliferation of cardiomyocytes. Few studies have focused on the molecular mechanisms through which changes in gravity induce phenotypic alterations of cardiomyocytes. Here, we show that calcium signalling was increased to a greater by hypergravity compared with microgravity, which may underlie the difference in extent of cardiomyocyte remodelling between these gravity states.

## CONFLICT OF INTEREST

The authors declare no commercial or financial conflict of interest.

## AUTHOR CONTRIBUTIONS

Caizhi Liu, Guohui Zhong, Yuezhang Zhou and Yuchen Yang performed the majority of the experiments, analysed data and prepared the paper. Yingjun Tan provided us technical support. Yuheng Li, Xingcheng Gao, Weijia Sun, Jianwei Li, Xiaoyan Jin, Dengchao Cao and Xinxin Yuan helped with simulated microgravity and hypergravity experiments. Zizhong Liu, Shuai Liang, Youyou Li, Ruikai Du, Yinlong Zhao, Jianqi Xue, Dingsheng Zhao and Jinping Song provided suggestions for the project and critically reviewed the paper. Shukuan Ling and Yingxian Li supervised the project and the paper.

## Data Availability

The data that support the findings of this study are available on request from the corresponding author (yingxianli@aliyun.com).
