# Supplementary material for: Alteration of calcium signalling in cardiomyocyte induced by simulated microgravity and hypergravity
Source: Cell Prolif. 2020 Feb 26;53(3):e12783. doi: 10.1111/cpr.12783 (PMC7106961; doi:10.1111/cpr.12783)
Supplement: Supplementary file 1 [file CPR-53-e12783-s001.docx]

Supplementary Fig.1


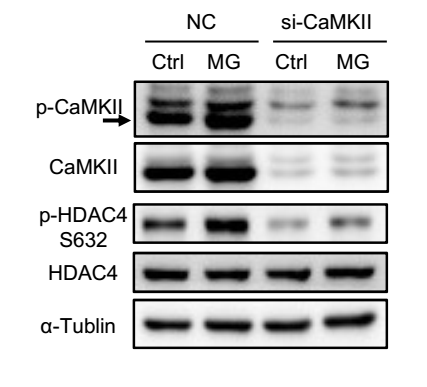
 A.

B.


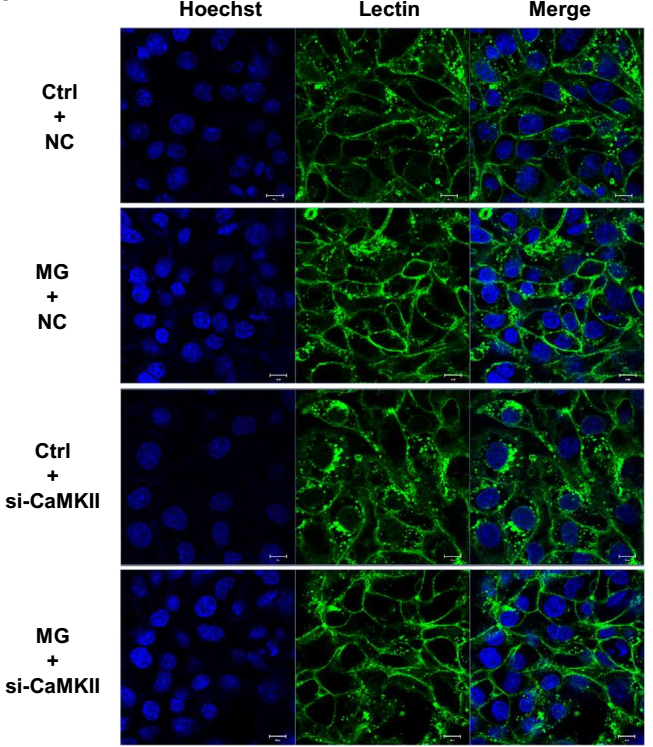


**Supplementary Figure 1. Effect of CaMKII signaling in rotation-simulated microgravity.**

A. Expression of p-CaMKII and p-HDAC4 treated with rotation-simulated microgravity after cells transfected with si-*CaMKII* or NC (negative control). (B) WGA staining of HL-1 cell to demarcate cell boundaries in each group. Scale bar: 10 μm.

Supplementary Fig.2


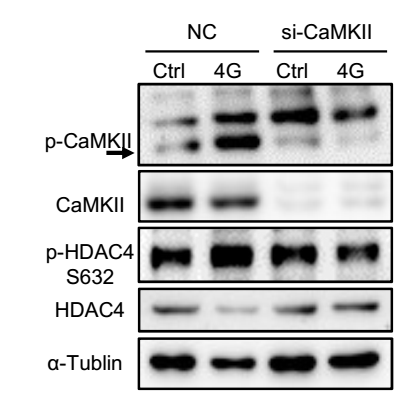


A.


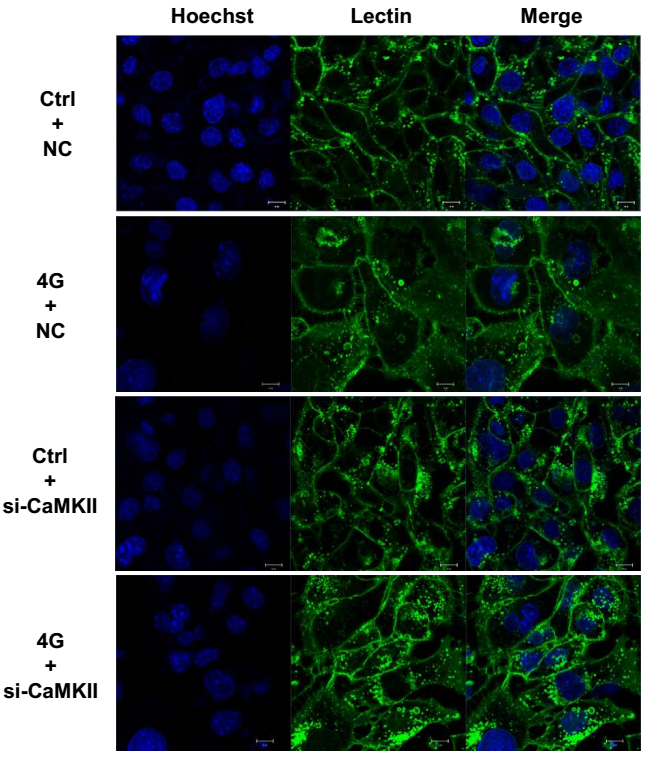
B.

**Supplementary Figure 2. Effect of CaMKII signaling in HL-1 cell treated with 4G.**

A. Expression of p-CaMKII and p-HDAC4 treated with 4G centrifugation after cells transfected with si-*CaMKII* or NC (negative control). B. WGA staining of HL-1 cell to demarcate cell boundaries in each group. Scale bar: 10 μm.
